# A thermostable and CBM2-linked GH10 xylanase from *Thermobifida fusca* for paper bleaching

**DOI:** 10.3389/fbioe.2022.939550

**Published:** 2022-08-26

**Authors:** Xiuyun Wu, Zelu Shi, Wenya Tian, Mengyu Liu, Shuxia Huang, Xinli Liu, Hua Yin, Lushan Wang

**Affiliations:** ^1^ State Key Laboratory of Biobased Material and Green Papermaking, Qilu University of Technology, Shandong Academy of Sciences, Jinan, China; ^2^ State Key Laboratory of Biological Fermentation Engineering of Beer, Qingdao, China; ^3^ State Key Laboratory of Microbial Technology, Institute of Microbial Technology, Shandong University, Qingdao, China

**Keywords:** xylanase, glycoside hydrolase family 10, carbohydrate-binding module, active-site architecture, site-directed mutation

## Abstract

Xylanases have the potential to be used as bio-deinking and bio-bleaching materials and their application will decrease the consumption of the chlorine-based chemicals currently used for this purpose. However, xylanases with specific properties could act effectively, such as having significant thermostability and alkali resistance, etc. In this study, we found that *Tf*Xyl10A, a xylanase from *Thermobifida fusca*, was greatly induced to transcript by microcrystalline cellulose (MCC) substrate. Biochemical characterization showed that *Tf*Xyl10A is optimally effective at temperature of 80 °C and pH of 9.0. After removing the carbohydrate-binding module (CBM) and linker regions, the optimum temperature of *Tf*Xyl10A-CD was reduced by 10°C (to 70°C), at which the enzyme’s temperature tolerance was also weakened. While truncating only the CBM domain (*Tf*Xyl10AdC) had no significant effect on its thermostability. Importantly, polysaccharide-binding experiment showed that the auxiliary domain CBM2 could specifically bind to cellulose substrates, which endowed xylanase *Tf*Xyl10A with the ability to degrade xylan surrounding cellulose. These results indicated that *Tf*Xyl10A might be an excellent candidate in bio-bleaching processes of paper industry. In addition, the features of active-site architecture of *Tf*Xyl10A in GH10 family were further analyzed. By mutating each residue at the -2 and -1 subsites to alanine, the binding force and enzyme activity of mutants were observably decreased. Interestingly, the mutant E51A, locating at the distal -3 subsite, exhibited 90% increase in relative activity compared with wild-type (WT) enzyme *Tf*Xyl10A-CD (the catalytic domain of *Tf*Xyl110A). This study explored the function of a GH10 xylanase containing a CBM2 domain and the contribution of amino acids in active-site architecture to catalytic activity. The results obtained provide guidance for the rational design of xylanases for industrial applications under high heat and alkali-based operating conditions, such as paper bleaching.

## Introduction

In recent years, enzymes have been widely used in the pulp and paper industry to ensure environmentally sustainable development, especially in the pulp bleaching process (Gupta et al., 2022). In the bleaching process, biological enzymes can not only reduce the amount of chemical bleaching agent needed to improve the whiteness of paper pulp, but also reduce the content of ionic waste in the bleaching liquid (Kumar et al., 2016; Gupta et al., 2022). Xylanase is a common bleaching agent, which can open the link between cellulose and lignin by degrading the xylan in plant fiber and facilitate the removal of lignin (Walia et al., 2017). Xylanase can improve the bleaching effect of pulp in elemental chlorine free bleaching (ECF) and total chlorine free bleaching (TCF) processes, and the physical properties of pulp can also be further improved (Rencoret et al., 2013). Saleem et al. (2009) reported 15% decrease in chlorine consumption, 26% increase in brightness and 16.2% reduction in kappa number for wood pulp pretreatment with thermostable xylanase from *Bacillus* Sp. XTR-10. In another study, the use of xylanase from *Aspergillus nidulans* with optimum temperature (60 °C) and pH (9.0) could reduce lignin content of bamboo pulp, as shown by the reduced peak intensity and the reduction in kappa number (Khambhaty et al., 2018). One of the limitations of xylanase application in bio-bleaching is that these enzymes should be both thermostable and alkaline-stable (Kumar et al., 2016; Talens-Perales et al., 2020). Previous studies have reported the application of a xylanase (XynG1-1R) from *Paenibacillus campinasensis* G1-1 (optimal activity at 60°C and pH 7.0) and xylanase A from *Bacillus halodurans* C-125 (optimal activity at 70°C and pH 9.0) on bio-bleaching of pulp and paper (Zheng et al., 2012; Lin et al., 2013). Thus, an increasing number of studies are focusing on the identification and generation of xylanases with higher thermal and alkali resistance (optimum temperature above 60°C, optimum pH above 7.0) and that this is a major biotechnological goal in the pulp and paper industry.

Xylanases (EC 3.2.1.8) are a group of enzymes that hydrolyze the β-1,4 bond in the xylan backbone. According to the CAZy database (http://www.cazy.org), xylanases are classified within a number of glycoside hydrolase (GH) families, with the vast majority of characterized xylanases belonging to GH10 and GH11 families (Lombard et al., 2013). The GH11 xylanases usually possess only a catalytic domain (Miao et al., 2017) and may contains secondary binding site on their surface (Ludwiczek et al., 2007). In contrast, most GH10 xylanases contain carbohydrate binding modules (CBMs) (Ali et al., 2005; Guillén et al., 2010). Recent studies have shown that CBMs apparently exhibit substrate specificities, such as CBM 1, 2a, 3, 5, and 10 have affinity for crystalline cellulose, while CBM 2b, 4, 6, 13, 15, and 22 specifically bind to xylan substrates (Boraston et al., 2004; Guillén et al., 2010). The catalytic activity of xylanase was increased by fusing XynB from *Thermotoga Maritima* with CBM2 (Kittur et al., 2003), while the addition of CBM6 and CBM22 to xylanase Z of *Clostridium thermocellum* resulted in decreased activity and facilitated activity, respectively (Khan et al., 2013). Meng et al. (2015)reported that CBM was crucial to enzymatic activity and thermostability of GH10 XynB from *Caldicellulosiruptor* sp. F32 through truncation experiments. In addition, since the active site architectures of GH10 and GH11 xylanases were different, substituted xylose residues could locate at +1 and −3 subsites in GH10 family xylanases but at the +2 subsite in GH11 family xylanases (Pell et al., 2004; Pollet et al., 2009; Gong et al., 2016).

*Thermobifida fusca*, a cellulolytic bacterium, possesses excellent physiological and biochemical characteristics, such as thermostability, high activity and wide pH tolerance (Gomez del Pulgar and Saadeddin, 2014). *T. fusca* can efficiently degrade cellulosic biomass by secreting a variety of glycoside hydrolases, such as cellulase, xylanase, arabinofuranosidase, mannanase, 1,3-β-glucanase (Gomez del Pulgar and Saadeddin, 2014). Among these enzymes, three xylanases have been discovered, namely Xyl10A and Xyl10B of the GH10 family and Xyl11A of the GH11 family. A previous functional proteomic study found that GH11 xylanase derived from *T. fusca* was mainly induced by xylan substrates, while GH10 xylanase was mainly induced by microcrystalline cellulose (MCC) substrate (Shi et al., 2020). Xyl10B and Xyl11A had been purified and characterized, and the latter was shown to act more effectively on xylan, although it was less active than Xyl10B on corn fiber (Kim et al., 2004; Zhao et al., 2015). In addition, three endoxylanases (Xyl10A, Xyl10B and Xyl11A) and one xylosidase (Xyl43A) from *T. fusca* were designed as an artificial cellulosome, which could effectively hydrolyze the wheat straw substrate (Morais et al., 2011). However, the function and characterization of Xyl10A have not been explored in detail. The CBM domain of Xyl10A could bind crystalline regions, helping GH10 xylanases to hydrolyze washed corncob particles (WCCP) rather than pure xylan (Inoue et al., 2015; Miao et al., 2017).

In the present study, we investigated the structure and function of a multi-domain xylanase (*Tf*Xyl10A) obtained from *T. fusca*, in order to exploit a candidate enzyme for bio-bleaching process. The domain truncation experiment was to explore the role of each domain of the enzyme. Moreover, the abilities to hydrolyze different pretreated corn stovers and bind to cellulose and xylan substrates were determined to elucidate the catalytic specificity of *Tf*Xyl10A and different truncated mutants. Structural bioinformatics analysis and an alanine-scanning experiment of the active-site architecture were performed to better comprehend structural basis of catalytic activity of GH10 xylanases. This study will contribute to understanding the function and catalytic mechanism of a thermo-alkali xylanase, and provide guidance for the rational design and industrial application of enzymes belonging to the GH10 family.

## Materials and methods

### Strains and cultivation conditions

*Thermobifida fusca* DSM43792 (Genebank accession no. AF002264) was purchased from Deutsche Sammlung von Mikroorganismen und Zellkulturen (DSMZ, Braunschweig, Germany). A 0.5% (v/v) inoculant was used for cells growth in Czapek’s medium at 55°C and the culture was shaken at 200 rpm for 20 h. The medium was centrifuged at ×5000 g for 2 min to collect cells, followed by washing two times using sterile water. Then, these cells were transformed into fresh Czapek’s mediums with 1% (w/v) of xylan or microcrystalline cellulose (MCC) as the sole induced carbon source. The cells were harvested at different sampling time (0, 2, 6, and 12 h) by centrifuging at ×5000 g for 2 min and then stored at −80°C.

*Escherichia coli* DH5α and BL21 (DE3) (Dingguo, Beijing, China) were used for molecular cloning and secretory expression of xylanases, respectively. All *E. coli* strains were grown in Luria-Bertani (LB). The Blunt-E1 plasmid (Transgen, Beijing, China) was used as the expression vector.

### RNA isolation and quantitative PCR

Bacteria samples at different time points were used to extract RNA and synthesize cDNA. Total RNA was extracted using the TRIzol method (Rio et al., 2010). And the integrity of RNA was confirmed by 1% (w/v) agarose gel electrophoresis. The cDNA was synthesized using an Evo M-MLV reverse transcription kit (AG, China) according to the manufacturer’s protocol. The concentration and purity of cDNA were determined by measuring UV absorbance with the Nanophotometer ^®^ N60 (Implen, Munich, Germany). The qPCR was carried out using power SYBR^®^ Green Pro Taq HS (AG, China) in qTOWER^3^G (Analytic Jena, Jena, Germany). The primers used to detect the transcriptional levels of encoding genes are listed in Supplementary Table S1. The target genes included three xylanase genes (*xyl11a*, AHK22788.1; *xyl10a*, AAZ56956.1; *xyl10b*, AAZ56824.1). The gyrase gene (*gyra*, AAZ54047.1) was used as an internal reference gene. The transcription of all target genes were normalized relative to the *gyra* gene. Three biological replicates and three experiment replicates were performed for each sample.

### Plasmid construction

The genes, encoding the *Tf*Xyl10A, *Tf*Xyl10AdC (catalytic domain + linker), and *Tf*Xyl10A-CD (only catalytic domain), were amplified from the genomic DNA of *T. fusca* DSM10635 and cloned into the vector Blunt-E1 to obtain recombinant plasmids. The gene encoding *Tf*Xyl10A-CD was used as the wild-type (WT) and site-directed mutagenesis was performed using a PCR-based method (Weiner et al., 1994). A PCR was performed (S1000 Thermal Cycler; Bio-Rad, Hercules, CA, United States) using PrimeSTAR Max Premix (TaKaRa, Shiga, Japan). The sequences of primers used for expression and mutagenesis are listed in Supplementary Table S2. After digesting with *Dpn*I (ABclonal Biotech, Wuhan, China) and fragment purification, the PCR product was transformed into *E. coli* DH5α using LB-ampicillin plates. Plasmid recovery was conducted using a GV-plasmid DNA mini extraction kit (Dingguo, Beijing, China) followed by sequencing (Tsingke, Qingdao, China) to verify the mutations. All constructed recombinant plasmids contained an N-terminal His_6_ tag.

### Protein expression and purification

The correct recombinant plasmids were further transformed into *E. coli* BL21 (DE3) to produce recombinant proteins induced by 1 mM isopropyl β-D-thiogalactopyranoside (IPTG, Sangon Biotech, Shanghai, China) for 20 h at 20°C. *E. coli* BL21 (DE3) cells were harvested by centrifugation and resuspended in pH 8.0 NaH_2_PO_4_/NaCl buffer (50 mM NaH_2_PO_4_ and 300 mM NaCl). Ultrasonic Homogenizer (JY92-IIDN, SCIENTZ, Ningbo, China) was used for cell fragmentation. The method was to use 20 kHz frequency, 200 W power, each crushing 30 s, 30 s interval, repeated crushing 30 min at 4°C. After ultrasonic fragmentation, the recombinant proteins were purified with Ni Sefinose™ 6 Fast Flow (GE Healthcare Bio-Science AB, Uppsala, Sweden). The samples were applied to the column by gravity flow. First, the column was equilibrated with 10 ml pH 8.0 NaH_2_PO_4_/NaCl buffer with 5 mM imidazole. Subsequently, after the sample has entered the resin, the column was washed with 20 ml of washing buffer (50 mM NaH_2_PO_4_ and 300 mM NaCl, 20 mM imidazole; pH 8.0) to remove nonspecifically bound proteins. Then, 10 ml of elution buffer (50 mM NaH_2_PO_4_ and 300 mM NaCl, 100 mM imidazole; pH 8.0) and 10 ml of elution buffer (50 mM NaH_2_PO_4_ and 300 mM NaCl, 250 mM imidazole; pH 8.0) were used to collect target proteins. The purified proteins were analyzed by sodium dodecyl sulfate-polyacrylamide gel electrophoresis (SDS-PAGE). The protein marker was enhanced 3-color regular range protein marker with the segments range of 10−180 KDa (PM2510, SMOBIO Technology, Inc.). The protein concentration was measured by the Bradford method with bovine serum albumin as the standard (Bradford, 1976).

### Enzymatic properties assays

Xylanase activity was measured by the dinitrosalicylic (DNS) acid method (Miller, 1959). For detection of the optimal temperature, 0.25 μM of the purified enzymes was mixed with 1% xylan at different temperatures ranging from 30°C to 90°C in sodium citrate buffer (pH 7.0) for 10 min. The optimal pH of xylanases was determined in the range of pH 3.0–11.0 (50 mM sodium citrate buffer, pH 3.0–8.0; 50 mM Glycine-NaOH buffer, pH 9.0–10.0; Na_2_HPO_4_-NaOH buffer, pH 11.0) using beechwood xylan (BX) substrate for 10 min at 80°C. Thermostability of xylanases was measured by evaluating the residual activity (Han et al., 2020) at the optimum pH 9.0 after preincubating for 60 min at different temperatures ranging from 30°C to 90°C. In addition, xylanases were preincubated at 80°C for different time intervals (0, 5, 15, 30, and 60 min) and the residual activities were also determined under the optimum reaction conditions (80°C and pH 9.0). And we detected pH stability of xylanases by assessing the residual activity under the optimum reaction conditions (80°C and pH 9.0) after incubation at pH 9.0 for 0, 5, 15, 30 and 60 min. After enzyme and substrate mixed reaction, 80 μL of DNS solution was added and then the solution was boiled for 10 min immediately to terminate the reaction. After adding 820 μL deionized water, the absorbance was detected at a wavelength of 550 nm by an ultraviolet spectrophotometer (Puyuan Instruments, Ltd., Shanghai, China). One unit of xylanase activity (U) was defined as the amount of enzyme (mg) that released 1 μmol of xylose per minute under the optimal assay conditions.

### Hydrolysis of pretreated corn stover

Corn stover was collected from Dezhou, Shandong Province, China. The PCS was prepared as previously reported with a slight modification (Wang et al., 2019). The cleaned corn stover was crushed by a knife mill and subsequently passed through a 30-mesh sieve. The collected corn stover was incubated with 15% of aqueous ammonia solution for 12 h at 60°C and washed with deionized water until its pH was neutral. After being oven-dried at 80°C to obtain a constant weight, the solids were used as the substrate named “PCS-1”. The solids were further degraded by *Tf*Xyl11A to remove long-chain xylan backbone and the resulting substrate with less xylan residue was named “PCS-2”.

Then, 0.5 μM of *Tf*Xyl10A, *Tf*Xyl10AdC or *Tf*Xyl10A-CD was mixed with each substrate. After incubation at 80°C for different time periods, the reducing sugar released from each substrate was detected by the DNS method. Three replicates were performed for each sample.

### Measurement of the polysaccharide-binding capacity

Polysaccharide-binding experiments were carried out to clarify the binding characteristics of the CBM2 domain in *Tf*Xyl10A. Two different natural polysaccharides, corncob xylan and MCC, were dissolved in pH 7.0 Na_2_HPO_4_-citric acid buffer, and were centrifuged at ×14,000 g for 5 min; then the supernatant was discarded. Insoluble solid substrates were collected after five washes. *Tf*Xyl10A, *Tf*Xyl10AdC or *Tf*Xyl10A-CD (400 μL 1 μM) were incubated with each substrate (0.02 g) at 4°C for 1 h. Then the supernatants were collected after ×14,000 g centrifugation for 10 min at 4°C. The residual enzyme activity in the supernatants was measured using BX as a substrate.

### Enzyme kinetics

Kinetic analysis of the catalytic reactions were performed under standard conditions using purified enzymes (100 μL) and various concentrations of BX substrates (0.2–10%, 500 μL) for 5 min at 80°C, then 400 μL DNS solution was added to terminate the reaction. The kinetic parameters were nonlinearly fitted based on the Michaelis-Menten equation using Prism 5.0 software (GraphPad, San Diego, CA, United States) (Motulsky, 2007).

### Structural bioinformatic analysis

The sequence and structure information of GH10 xylanases were obtained from the National Center for Biotechnology Information (NCBI, http://www.ncbi.nlm.nih.gov/), Uniprot (http://www.uniprot.org/), Protein Data Bank (PDB, http://www.rcsb.org/), and Carbohydrate-Active enzyme (CAZy, http://www.cazy.org/Glycoside-Hydrolases.html). Swiss-model (http://beta.swissmodel.expasy.org/) was used to model the 3-D structure of *Tf*Xyl10A catalytic domain with the template of *Cf*Xyn10A (PDB 3CUJ). The xylanase *Cf*Xyn10A (PDB 3CUJ) were used to find the amino acid residues in the active-site architecture of GH10 xylanases. The conservation of the sequence profile was tested by the ConSurf Sever (Haim et al., 2016).

## Results

### Transcription of the *Tf*Xyl10A encoding gene induced by microcrystalline cellulose

**FIGURE 1:**
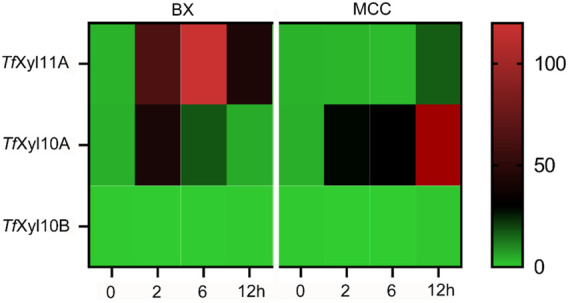
Transcript levels of genes encoding xylanases in *T. fusca* DSM10635 induced by beechwood xylan (BX) and microcrystalline cellulose (MCC) at different incubation times (0, 2, 6, and 12 h). Transcripts of all target genes were normalized relative to the *gyra* gene.

The genome of *T. fusca* DSM10635 contains three xylanase genes encoding one GH11 xylanase named *Tf*Xyl11A, and two GH10 xylanases named *Tf*Xyl10A and *Tf*Xyl10B. The transcription levels of the three xylanase genes were determined by qPCR with BX or MCC as the sole carbon source. As shown in Figure 1, both genes encoding *Tf*Xyl11A and *Tf*Xyl10A could be induced by BX substrate. Gene encoding *Tf*Xyl11A showed the highest level of transcription on BX at 6 h. While the amount of *Tf*Xyl10A transcription increased slightly at 2 h, and then appeared to decrease. However, the transcription level of the gene encoding *Tf*Xyl10A was lower overall than that of the gene encoding *Tf*Xyl11A. This might indicate that BX is not the best substrate for inducing to express *Tf*Xyl10A. Interestingly, the gene encoding *Tf*Xyl10A also had high transcription levels under MCC induction, with the transcription levels at 12 h being the highest (Figure 1). In addition, the gene encoding *Tf*Xyl10B was not expressed under either BX or MCC induction, suggesting that it might be a redundant xylanase gene. These results were consistent with previous proteome studies that identified two major xylanases *Tf*Xyl11A and *Tf*Xyl10A (Shi et al., 2020).

### Heterologous expression and enzymatic characterization of *Tf*Xyl10A and the truncation mutants (*Tf*Xyl10AdC and *Tf*Xyl10A-CD)

**FIGURE 2:**
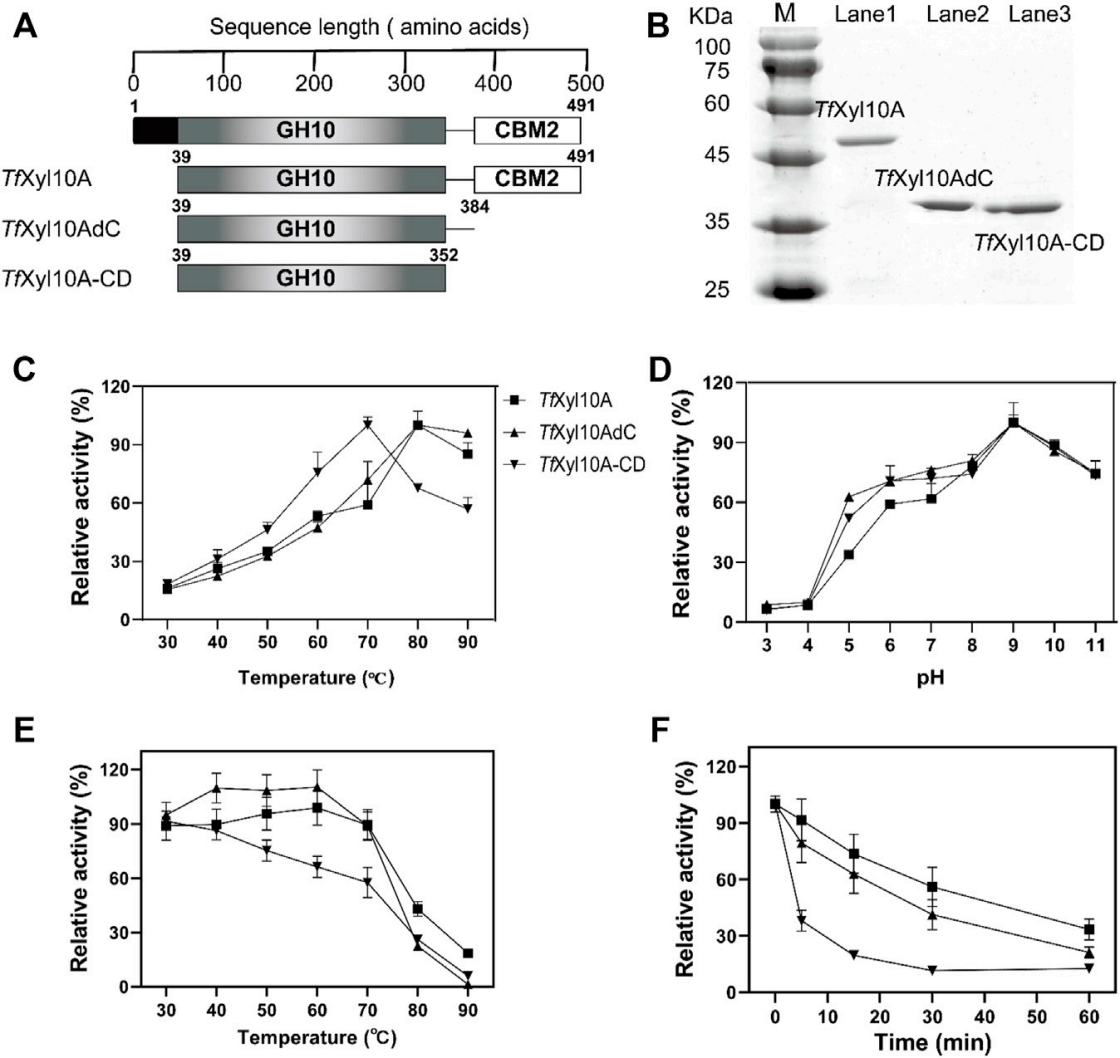
Schematic structures and biochemical characterization of *Tf*Xyl10A, *Tf*Xyl10AdC, and *Tf*Xyl10A-CD. **(A)** Schematic structural domain of *Tf*Xyl10A and the domain deletion mutants (*Tf*Xyl10AdC and *Tf*Xyl10A-CD). **(B)** SDS-PAGE of *Tf*Xyl10A, *Tf*Xyl10AdC, and *Tf*Xyl10A-CD. **(C)** Determination of optimum temperatures for the three xylanases. **(D)** Determination of optimum pH for the three xylanases. **(E)** Measurement of thermostability through the evaluation of the residual activity after the enzymes were preincubated for 60 min at different temperatures. **(F)** Kinetics of thermal inactivation of xylanases at 80°C under different time points. The activity of the unheated sample was set to 100%. The bars indicate the standard errors.

According to the descriptions in the uniprot database, the complete *Tf*Xyl10A contained a total of 491 amino acids, including a signal peptide (1–38 amino acids), catalytic domain (CD, 39–352 amino acids) and CBM2 domain (384–491 amino acids). The full-length *Tf*Xyl10A xylanase and truncation mutants (*Tf*Xyl10AdC and *Tf*Xyl10A-CD) were constructed (Figure 2A) and heterologously expressed using the *E. coli* expression system. The purified proteins were examined by SDS-PAGE (Figure 2B). The effects of temperature and pH on enzymatic activity were evaluated using BX as the substrate. As shown in Figure 2C, the optimum temperature of the xylanases *Tf*Xyl10A and *Tf*Xyl10AdC was 80°C and more than 80% of the activities were retained at 90°C, while the optimum temperature of *Tf*Xyl10A-CD was 10°C lower than that of *Tf*Xyl10A and *Tf*Xyl10AdC. The thermostability assay showed that *Tf*Xyl10A and *Tf*Xyl10AdC preserved more than 88% of the enzyme activity when preincubated at 30–70°C for 60 min, while *Tf*Xyl10A-CD preserved only 57% at 70°C (Figure 2E). In addition, *Tf*Xyl10A-CD incubated at 80°C for 5 min and the enzyme activity was significantly reduced to 38% of the original level (Figure 2F). These results indicated that the linker region is important to maintain the stability of the enzyme structure at high temperatures, but not the CBM domain. The maximum activity of *Tf*Xyl10A, *Tf*Xyl10AdC and *Tf*Xyl10A-CD occurred at pH 9.0 (Figure 2D), with 84% of the relative activity retention under of 9.0 for 60 min incubation (Supplementary Figure S1). These results suggested that *Tf*Xyl10A is a thermostable and alkaline xylanase, which could be an excellent candidate in industrial applications.

### Binding and degradation capacities of *Tf*Xyl10A and truncated mutants on xylan and cellulose

**FIGURE 3:**
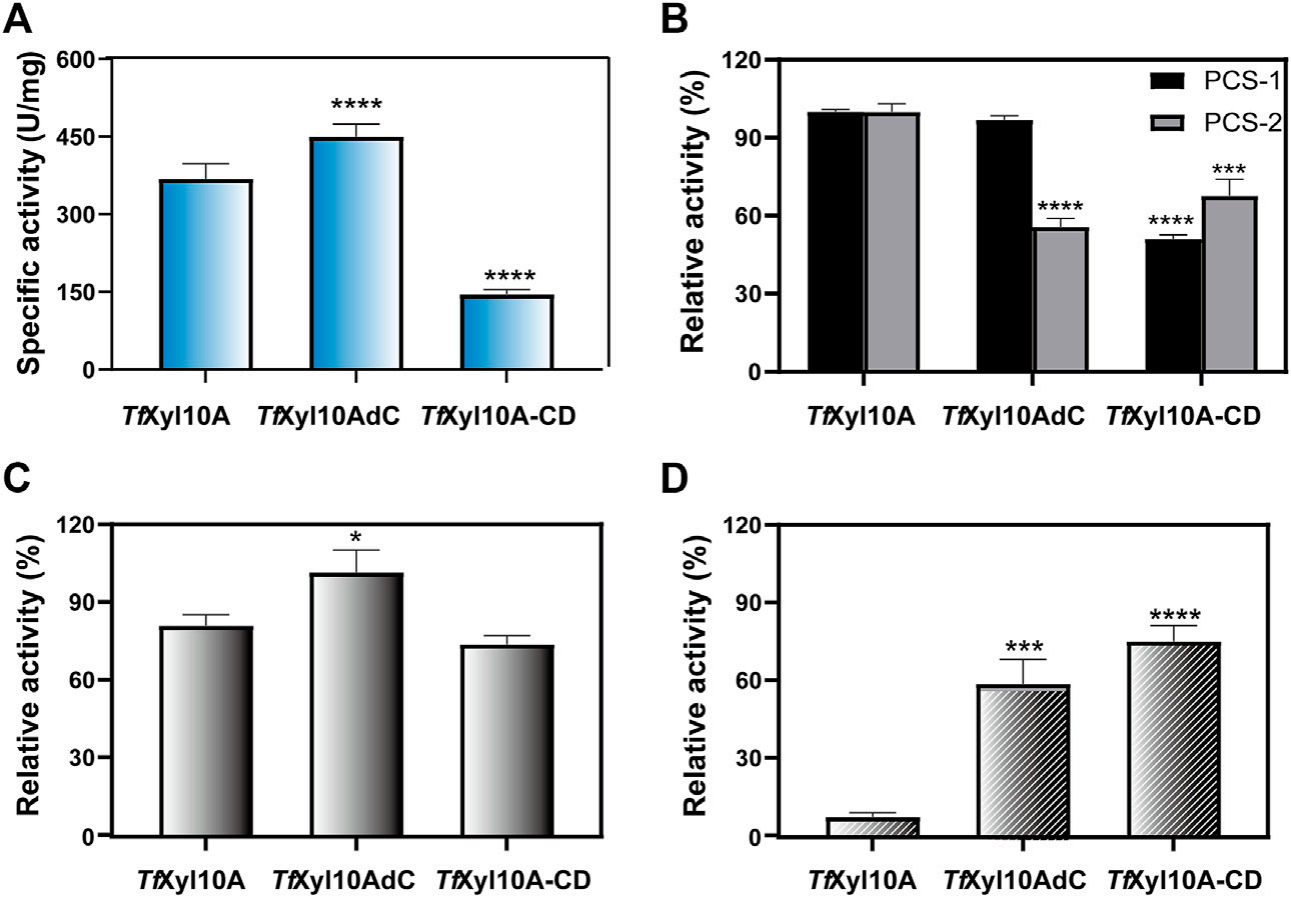
Enzyme activities and substrate binding capacities of *Tf*Xyl10A and the truncation mutants (*Tf*Xyl10AdC and *Tf*Xyl10A-CD). **(A)** Specific activity (U/mg) of *Tf*Xyl10A, *Tf*Xyl10AdC, and *Tf*Xyl10A-CD on beechwood xylan (BX). **(B)** Relative activity (%) of *Tf*Xyl10A, *Tf*Xyl10AdC, and *Tf*Xyl10A-CD on pretreated corn stover (PCS-1: pretreatment by 15% aqueous ammonia solution; PCS-2: pretreatment by 15% aqueous ammonia solution and hydrolysis of *Tf*Xyl11A). Enzymatic activity of *Tf*Xyl10A was defined as 100%. **(C)** Residual enzyme activities of the supernatants of *Tf*Xyl10A, *Tf*Xyl10AdC, and *Tf*Xyl10A-CD after binding with insoluble solid xylan substrate at 4°C for 1 h. **(D)** Residual enzyme activities of the supernatants of *Tf*Xyl10A, *Tf*Xyl10AdC, and *Tf*Xyl10A-CD after binding with microcrystalline cellulose (MCC) at 4°C for 1 h. The Enzymatic activities of three untreated enzymes was defined as 100%. The bars indicate the standard errors. Three biologically independent replicates were used to calculate means and standard deviations. *p*-values were calculated using Prism by performing one-way ANOVA, with a statistical significance level defined as ∗ (*p* < 0.05),∗∗ (*p* < 0.01),∗∗∗ (*p* < 0.001), and **** (*p* < 0.0001). Sequence alignment and structural analysis of *Tf*Xyl10A-CD.

The specific activity of the recombination enzyme *Tf*Xyl10A was 368.86 ± 28.85 U/mg on BX. *Tf*Xyl10AdC had a higher degradation ability than *Tf*Xyl10A on BX, while the enzyme activity of the mutant *Tf*Xyl10A-CD was the lowest (Figure 3A). This was consistent with previous reports of GH10 xylanases from *Aspergillus fumigatus* Z5, that is, Xyn10AdC, a CBM1-removing mutant, has been reported to have a higher ability to degrade xylan than Xyn10A (Miao et al., 2017). In the degradation of xylan, the linker of *Tf*Xyl10A was essential for enzyme activity. In addition, the three enzymes showed no significant activities against MCC (data not shown). The enzyme activities of *Tf*Xyl10A, *Tf*Xyl10AdC, and *Tf*Xyl10A-CD were measured using different pretreated corn stover (PCS) samples as substrates (Figure 3B). PCS-1, in which lignin was effectively removed by aqueous ammonia treating (Yoo et al., 2015), mainly contained cellulose and hemicellulose components. PCS-2 was PCS-1 dried sample treated with adequate enzymatic hydrolysis of GH11 family xylanase *Tf*Xyl11A. PCS-2, in which a large amount of long-chain xylan backbone was effectively degraded (Gong et al., 2016), contained cellulose and xylan residues attached to cellulose surface. The relative activities of *Tf*Xyl10A and *Tf*Xyl10AdC were similar, and the relative enzyme activity of *Tf*Xyl10A-CD was only 50% of the former on PCS-1. This suggested that deletion of both the CBM and linker region severely affects enzyme activity when xylan substrates are abundant, compared with deletion of the CBM domain alone. However, the relative enzyme activity of *Tf*Xyl10AdC and *Tf*Xyl10A-CD was significantly lower than that of *Tf*Xyl10A, when PCS-2 was used as a substrate. These results indicated that *Tf*Xyl10A could effectively degrade the xylan residues attached to the surface of straw, and CBM2 played an important role. The specific activities of *Tf*Xyl10A, *Tf*Xyl10AdC and *Tf*Xyl10A-CD are shown in Supplementary Table S3.

The substrate affinities of the three enzymes were determined through residual activities analysis after binding. As shown in Figure 3C, after binding with insoluble xylan, the relative residual activity of *Tf*Xyl10A and *Tf*Xyl10A-CD on BX decreased by 21.69% and 27.60% compared with their initial enzyme activity and that of *Tf*Xyl10AdC was almost unchanged, which indicated that compared with *Tf*Xyl10AdC, *Tf*Xyl10A and *Tf*Xyl10A-CD were more tightly bound to insoluble xylan. However, the binding capabilities to MCC were completely different (Figure 3D). *Tf*Xyl10A supernatant showed the lowest relative residual activity with losing all enzyme activity on BX, compared with *Tf*Xyl10AdC and *Tf*Xyl10A-CD. The relative residual activity of supernatants of *Tf*Xyl10AdC *and Tf*Xyl10A-CD on BX retained 59.34% and 69.48% of their initial enzyme activity, respectively. *Tf*Xyl10A containing the CBM2 domain could efficiently bind MCC, indicating that the CBM2 domain played an important role in terms of protein binding to the cellulose substrate. The residual activities of supernatant of *Tf*Xyl10A, *Tf*Xyl10AdC, and *Tf*Xyl10A-CD after binding with insoluble xylan and MCC are shown in Supplementary Table S4.

**FIGURE 4:**
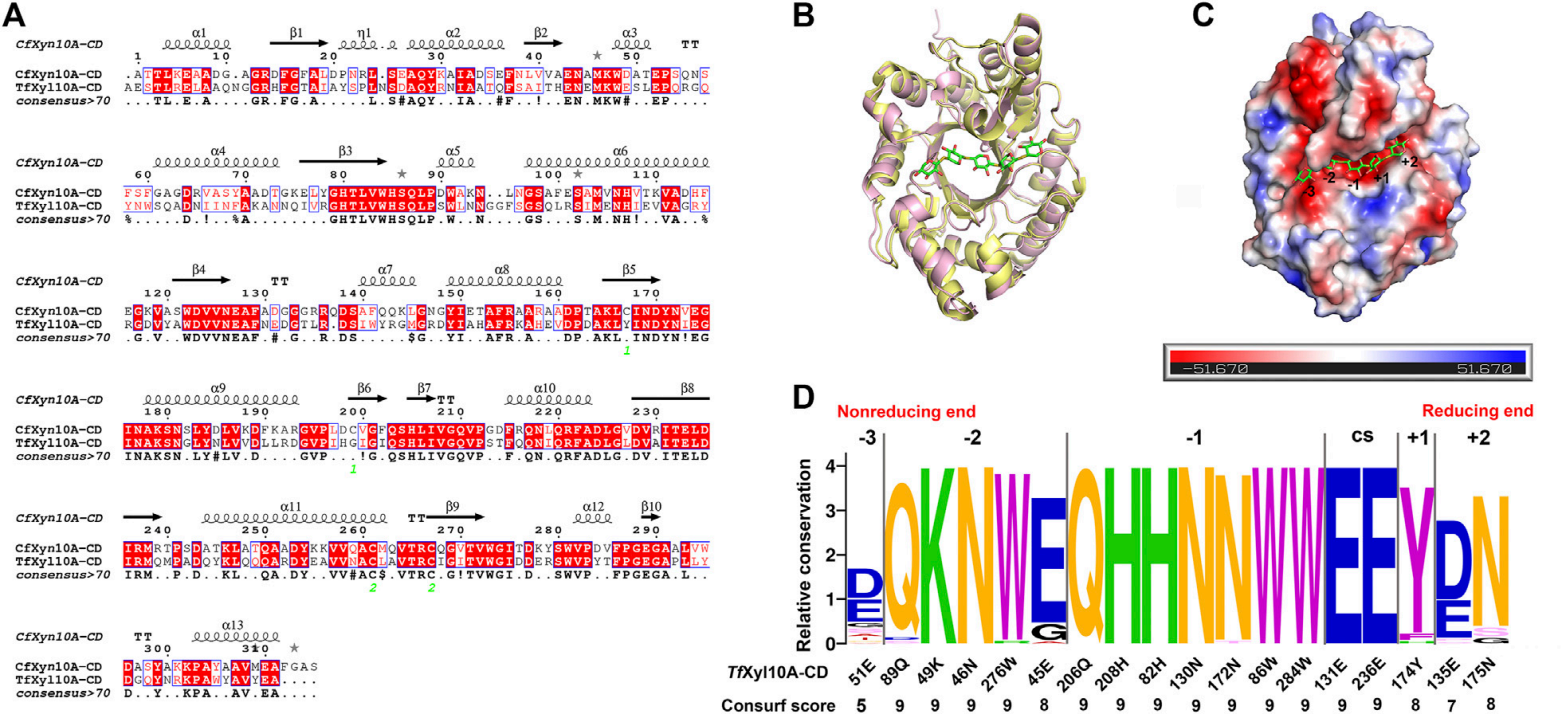
Structural analysis of *Tf*Xyl10A-CD. **(A)** Alignment of the GH10 domain of *Tf*Xyl10A with the protein used as a template for homology building (*Cf*Xyn10A, PDB: 3CUJ). The highlighted amino acids are identical or similar, and they are colored according to their physicochemical proprieties. Strictly conserved residues are highlighted by a red background, and conservatively substituted residues are boxed. The secondary structure of *Cf*Xyn10A is shown above the aligned sequences. **(B)** Superposition of the two proteins with the active cleft facing forward. *Cf*Xyn10A is shown in paleyellow, *Tf*Xyl10A-CD is shown in lightpink. **(C)** Surface representation of *Tf*Xyl10A-CD showing the fit of the xylopentaose in its active cleft and highlighting the −3, −2, −1, +1 and +2 subsites. **(D)** Sequence profile of the active site architecture of the GH10 family. The size of the letter and the consurf scores indicate the conservation degree of amino acids in each subsite. CS stands for cleavage site. Contributions of amino acid residues in the active site of *Tf*Xyl10A-CD.

To better comprehend structural basis of *Tf*Xyl10A-CD enzymatic activity, we built a 3-D homology model of the enzyme. *Cf*Xyn10A-CD xylanase from *Cellulomonas fimi* ATCC 484 (PDB: 3CUJ) was used as a structural template. The sequence alignment of the two proteins is given in Figure 4A, showing a sequence similarity of 54.69%. The conserved regions of the sequences made use of a color code. This conservation was directly reflected in the similarities of their tertiary structures. The two enzymes had a typical TIM α/β-barrel fold with a root-mean-square deviation (RMSD) of 0.549. (Figure 4B). *Cf*Xyn10A-CD with a sulfur substituted beta-1,4-xylopentaose was also used for a detailed structural analysis of the *Tf*Xyl10A-CD active site. *Tf*Xyl10A-CD showed a fit with xylopentaose in its active site and the −3, −2, −1, +1 and +2 subsites were highlighted (Figure 4C). The sequence profile of the active-site architecture of the GH10 family is shown in Figure 4D, with five and seven amino acid residues centered at the −2 and −1 subsites, respectively. Most of them were conserved across the GH10 family xylanases. There was a total of only four residues located at the subsites −3, +1, and +2. The consurf scores of 51E at −3 subsite was the lowest, “5”.

**FIGURE 5:**
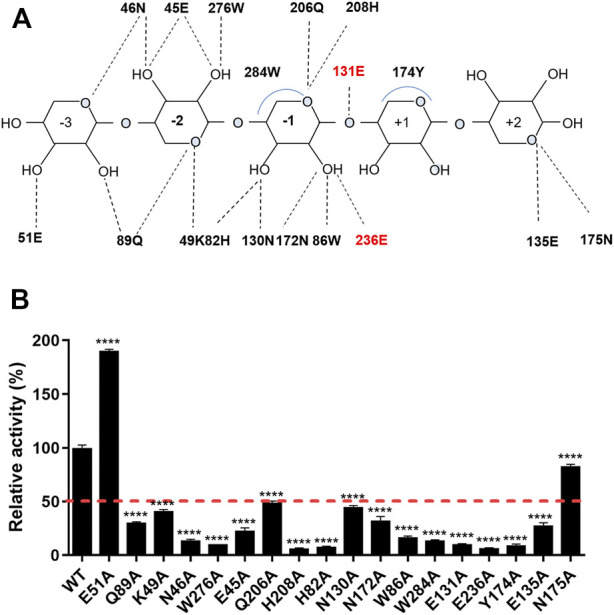
Schematic illustration of the interactions and enzymatic activities of the key residues in the active site of *Tf*Xyl10A-CD. **(A)** Schematic representation of the hydrogen bonding and hydrophobic stacking interactions in the binding cleft of *Tf*Xyl10A-CD with xylopentaose. Amino acids within a range of 5 Å of the ligand were selected to construct the active site architecture of enzyme. When the distance is less than 5 Å, the hydrogen bonding and hydrophobic stacking interactions might be formed between enzyme and ligand. Based on the structure display in Pymol, the schematic of the interactions was made. The two catalytic residues (131E and 236E) are highlighted in red. **(B)** Contributions of amino acid residues in the binding cleft to the catalysis of xylan. The release of reducing sugars in the reaction mixture was measured using the DNS method. The enzymatic activity of WT (*Tf*Xyl10A-CD) was set to 100%. The bars indicate the standard errors. *p*-values were calculated using Prism by performing one-way ANOVA, with a statistical significance level definded as ∗ (*p* < 0.05), ∗∗ (*p* < 0.01), ∗∗∗ (*p* < 0.001), and **** (*p* < 0.0001).

The interaction network between the amino acids of the *Tf*Xyl10A-CD active site and xyloheptaose was described in Figure 5A. To study the role of active-site residues in the binding of xylopentaose, we mutated the residues in the active site to alanine, and the relative activities of the mutants against BX were determined (Figure 5B). The residues Asn-46, Trp-276, His-208, His-82, Trp-86, Glu-131, and Glu-236 were involved in the hydrogen bonding with xylose at subsites −2, −1, and CS, and Trp-284 and Tyr-174 interacted with xylose at subsites −1 and +1 by hydrophobic stacking. This was critical for xylan hydrolysis because the alanine mutants of these residues retained only 10–20% of their activity. The results collectively indicated that the interaction for xyloses at the −2, −1, and +1 subsites were extremely important for xylan catalysis. Only Tyr-174 formed a CH-π hydrophobic interaction with the xylose ring at the +1 subsite, and the O2 and O3 positions of xylose could connect substituents (Figure 5A). The residues Gln-89, Lys-49, and Glu-45 at the −2 subsite and Gln-206, Asn-130 and Asn-172 at the −1 subsite formed a hydrogen bond with xylose, whose mutation to alanine led to approximately half-residual activities. Manipulating the residue Asn-175 at the +2 subsite to alanine also had a slight influence on the enzyme activity of *Tf*Xyl10A-CD. These results indicated that the interactions with a moderate importance in xylan hydrolysis could be at the subsite distal from the cleavage site. Surprisingly, the relative activity of mutant E51A at the −3 subsite was improved by 90% compared with that of the WT (Figure 5B). The specific activities of WT (*Tf*Xyl10A-CD) and its variants are shown in Supplementary Table S5. A structural analysis showed that Glu-51 might form an electrostatic interaction with xylose at the −3 subsite, but the interaction force of alanine disappeared after mutation (Supplementary Figure S2). This analysis was consistent with an increase in the kinetic constant *k*_cat_, suggesting that the mutant E51A might enhance the product releasing ability to improve the enzymatic activity (Table 1).

**TABLE 1:** Kinetic parameters of WT and the mutant E51A on BX.

Enzyme	*K*_m_ (mg/ml)	*k*_cat_ (1/s)	*k*_cat_/*K*_m_ (mL* s^−1^ *mg^−1^)
WT	4.29 ± 0.39	169 ± 2	39.58 ± 3.22
E51A	16.35 ± 0.86^**^	581 ± 11^***^	35.58 ± 1.20^ns^

P-values were calculated using Prism by performing one-way ANOVA, with a statistical significance level defined as ns (*p* > 0.05), ^∗^(*p* < 0.05), ^∗∗^(*p* < 0.01), ^∗∗∗^(*p* < 0.001), and ^****^(*p* < 0.0001).

## Discussion

In this study, we found that one xylanase *Tf*Xyl10A in *T. fusca* could be induced by the cellulose substrate MCC, while the other two xylanases (*Tf*Xyl11A and *Tf*Xyl10B) were not (Figure 1). Previous studies have shown that cellobiose could induce the expression of cellulases, cellulose-binding proteins, and a xylanase *Tf*Xyl10A (Chen and Wilson, 2007). Deng and Fong (2010) measured the cellulase-related gene expression in WT and *celR*-disrupted *T. fusca* and found that there were no detectable mRNA transcripts of *Tf*Xyl10A in any of the samples of the *celR* deletion strain, confirming that CelR may activate transcription of this gene. The biochemical experiments showed that *Tf*Xyl10A containing the CBM2 domain could efficiently hydrolyze PCS-2 (Figure 3B) and bind to the MCC substrate (Figures 3C,D), indicating that this CBM had an important role in terms of protein binding to the cellulose substrate. The CBM1 is known to specifically bind to insoluble and highly crystalline cellulose and/or chitin (Gilbert et al., 2013). In a previous study, we also identified a GH10 family xylanase with CBM1 (G0SBF1), which was co-expressed with most cellulolytic enzymes in temporal secretomes and a transcriptional analysis of *Chaetomium thermophilum* (Li et al., 2020)*.* CBMs can potentiate the action of a cognate catalytic module toward polysaccharides in intact cell walls through the recognition of nonsubstrate polysaccharides, which explains why cellulose-directed CBMs are appended to many noncellulases (Herve et al., 2010; Inoue et al., 2015; Miao et al., 2017). *Tf*Xyl10A with CBM2 might be responsible for removing the residual xylan attached to cellulose, thus clearing the way for cellulase action.

Xylanase application in biobleaching could remove the xylan cross-linking layer within pulp fibers, but require alkaline cooking at high temperature (Kumar et al., 2016; Talens-Perales et al., 2020). Thermostable and alkali-tolerant enzymes can also be advantageous for other industrial productions because the reactions can be performed at an elevated temperature and the structure of the enzymes is stabilized in these extreme conditions (Kumar et al., 2016; Mhiri et al., 2020; Zarafeta et al., 2020). *T. fusca* is a thermophilic actinobacterium that grows at 50–55°C and lives in a wide pH range from 4.0 to 10.0 (Lykidis et al., 2007). The optimal temperature of *Tf*Xyl10A is 80°C (Figure 2C) and the optimal pH of *Tf*Xyl10A is 9.0 (Figure 2D). The optimal temperature was higher than that of most fungal xylanases (40°C–70°C) (Polizeli et al., 2005). Of course, there are many natural xylanases with superior properties to *Tf*Xyl10A. For example, a thermophilic GH10 xylanase XynAF1 from the high-temperature composting strain *Aspergillus fumigatus* Z5 has optimal reaction temperature of 90°C and the thermostability of its mutant XynAF1-AC was increased by 6-fold through semi-rational design (Li et al., 2021). Xyn11 from *Pseudothermotoga thermarum* showed an excellent activity at 90°C and pH 10.5, and the fusion of CBM2 to Xyn11 enhanced the enzymatic activity under extreme conditions (Talens-Perales et al., 2021). Therefore, the catalytic efficiency and thermostability of *Tf*Xyl10A could be further improved by protein engineering. However, in this study, we found that the removal of CBM2 did not affect the optimal temperature and pH of the enzyme (Figures 2C,D), but it slightly increased the enzyme activity (Figure 3A). The removal of the linker had a great influence on both enzyme activity and thermal stability (Figures 2C, 3A). The linker region contains 9 Pro and 8 polar amino acids (2 Asp + 3 Glu + 2 Ser + 1 Thr), more than half of the total number of amino acids, which play an important role in structural stability (Talens-Perales et al., 2021). The deletion of the linker region may allow to deconstruct more easily the catalytic domain from the C-terminal under elevated temperatures. Previous studies have indicated that the linker length and flexibility play an important role in the activities of some cellulases, such as BsCel5A from *Bacillus subtilis* (Ruiz et al., 2016) and PoCel6A from *Penicillium oxalicum* (Gao et al., 2015). The linker region promotes enzyme activity and binding efficiency of LPMO towards polymeric substrate (Srivastava et al., 2022). In addition, Sonan et al. (2007) have reported that the linker region plays a key role in the cold adaptation of the a cellulase from an *Antarctic bacterium*.

Previous studies have compared the ligand-binding promiscuity and specificity of the main GH families by analyzing the active-site architecture (Liu et al., 2015; Tian et al., 2016). The active-site architecture analysis of the GH10 family revealed that 14 of 18 residues were well-conserved (consurf scores were “9”) across the active cleft (Figure 4D), which might be the most important for xylan degradation. Except for catalytic residues, conserved amino acids are mainly concentrated at the −2 and −1 subsites (Figure 4D). Interaction network analysis showed that the subsites −2 and −1 may provide main hydrogen-bonding for GH10 xylanases (Figure 5A). It was not surprising that most of the mutants had an impaired activity because the substrate-binding residues are relatively conserved and are important for enzyme functions. The amino acid residues located in the substrate active-site architecture of glycoside hydrolases, especially polar amino acids, and aromatic residues, played important roles in substrate recognition, catalysis, and products release (Chu et al., 2017; Yang and Han, 2018). Moreover, only one aromatic amino acid, Tyr, acted with xylose residues at the +1 subsite, which may be the structural basis for accommodating substituted xylose residues in this subsite, consistent with the previous analysis of the crystalline structure of GH10 xylanase (Pell et al., 2004). In another study, MeGlcA^2^Xyl_3_ was the shortest side-chain hydrolysates for GH10 family xylanases, which also indicated the ability of +1 and −3 subsites on the xylanases to accommodate substituted xylose residues (Gong et al., 2016). Surprisingly, the relative activity of the mutant E51A at the −3 subsite was about 90% higher than that of the WT (Figure 5B). Although the catalytic efficiency (*k*_cat_/*K*_m_) of E51A was slightly lower than WT, the mutation of Glu reduced the binding force between enzyme and substrate. The enhancement of enzyme activity may be caused by the increase of product release rate. This was consistent with the results of previous studies that manipulated the residues interacting with substrates in the distal regions could improve the activities of xylanases from GH11 families (Wu et al., 2018; Wu et al., 2020). To study the molecular mechanism underlying the catalytic promiscuity of *Cb*Xyn10C, Chu et al. (2017) found that Glu-52 at the −3 subsite had an important effect on enzyme activity, and mutation into many residues resulted in nearly doubled activity on cellulose with no significant change in xylanase activity.

To conclude, this study explored the function and molecular basis of a GH10 xylanase *Tf*Xyl10A, which was induced by a cellulose substrate. *Tf*Xyl10A is a thermostable and alkali-tolerant xylanase. *Tf*Xyl10A had a strong removal ability for substituted xylan residues attached to inner cellulose layers, and CBM2 could help *Tf*Xyl10A to bind to cellulose. These results suggested that *Tf*Xyl10A with CBM2 domain presented more advantages for industrial applications compared to the other enzymes. In addition, active-site architecture analysis and mutation experiments showed that the most important residues were centered on the −2 and −1 subsites near the cleavage site, whereas residues with moderate roles could be located at more distal regions of the binding cleft. Manipulating the amino acid residue at the distal −3 subsite of the active-site architecture of GH10 enzymes could improve their enzymatic activities. The results of this study not only identified an excellent candidate for bio-bleaching, but also provided guidance for the rational design and industrial application of xylanases belonging to the GH10 family. Further studies are required to create novel xylanases with higher thermal and alkali resistance and develop lignocellulosic hydrolysis methods to improve the efficiency of the catalytic process and increase techno-economic feasibility.

## Data Availability

The original contributions presented in the study are included in the article/Supplementary Material, further inquiries can be directed to the corresponding authors.

## References

[B1] Ali E. Araki R. Zhao G. Sakka M. Karita S. Kimura T. (2005). Functions of family-22 carbohydrate-binding modules in *Clostridium josui* Xyn10A. Biosci. Biotechnol. Biochem. 69 (12), 2389–2394. 10.1271/bbb.69.2389 16377898

[B2] Boraston A. B. Bolam D. N. Gilbert H. J. Davies G. J. (2004). Carbohydrate-binding modules: fine-tuning polysaccharide recognition. Biochem. J. 382, 769–781. 10.1042/bj20040892 15214846 PMC1133952

[B3] Bradford M. M. (1976). A rapid and sensitive method for the quantitation of microgram quantities of protein utilizing the principle of protein-dye binding. Anal. Biochem. 72 (1), 248–254. 10.1016/0003-2697(76)90527-3 942051

[B4] Chen S. Wilson D. B. (2007). Proteomic and transcriptomic analysis of extracellular proteins and mRNA levels in *Thermobifida fusca* grown on cellobiose and glucose. J. Bacteriol. 189 (17), 6260–6265. 10.1128/JB.00584-07 17601791 PMC1951905

[B5] Chu Y. Tu T. Penttinen L. Xue X. Wang X. Yi Z. (2017). Insights into the roles of non-catalytic residues in the active site of a GH10 xylanase with activity on cellulose. J. Biol. Chem. 292 (47), 19315–19327. 10.1074/jbc.M117.807768 28974575 PMC5702671

[B6] Deng Y. Fong S. S. (2010). Development and application of a PCR-targeted gene disruption method for studying CelR function in *Thermobifida fusca* . Appl. Environ. Microbiol. 76 (7), 2098–2106. 10.1128/AEM.02626-09 20097808 PMC2849239

[B7] Gao L. Wang L. Jiang X. Qu Y. (2015). Linker length and flexibility induces new cellobiohydrolase activity of PoCel6A from *Penicillium oxalicum* . Biotechnol. J. 10 (6), 899–904. 10.1002/biot.201400734 25866282

[B8] Gilbert H. J. Knox J. P. Boraston A. B. (2013). Advances in understanding the molecular basis of plant cell wall polysaccharide recognition by carbohydrate-binding modules. Curr. Opin. Struct. Biol. 23 (5), 669–677. 10.1016/j.sbi.2013.05.005 23769966

[B9] Gomez del Pulgar E. M. Saadeddin A. (2014). The cellulolytic system of *Thermobifida fusca* . Crit. Rev. Microbiol. 40 (3), 236–247. 10.3109/1040841X.2013.776512 23537325

[B10] Gong W. Zhang H. Tian L. Liu S. Wu X. Li F. (2016). Determination of the modes of action and synergies of xylanases by analysis of xylooligosaccharide profiles over time using fluorescence-assisted carbohydrate electrophoresis. Electrophoresis 37 (12), 1640–1650. 10.1002/elps.201600041 27060349

[B11] Guillén D. Sánchez S. Rodríguez-Sanoja R. (2010). Carbohydrate-binding domains: multiplicity of biological roles. Appl. Microbiol. Biotechnol. 85 (5), 1241–1249. 10.1007/s00253-009-2331-y 19908036

[B12] Gupta G. K. Dixit M. Kapoor R. K. Shukla P. (2022). Xylanolytic enzymes in pulp and paper industry: new technologies and perspectives. Mol. Biotechnol. 64 (2), 130–143. 10.1007/s12033-021-00396-7 34580813

[B13] Haim A. Shiran A. Eric M. Ofer C. Itay M. Tal P. (2016). ConSurf 2016: an improved methodology to estimate and visualize evolutionary conservation in macromolecules. Nuclc Acids Res. 44 (W1), W344–W350. 10.1093/nar/gkw408 PMC4987940 27166375

[B14] Han C. Liu Y. Liu M. Wang S. Wang Q. (2020). Improving the thermostability of a thermostable endoglucanase from *Chaetomium thermophilum* by engineering the conserved noncatalytic residue and N-glycosylation site. Int. J. Biol. Macromol. 164, 3361–3368. 10.1016/j.ijbiomac.2020.08.225 32888988

[B15] Herve C. Rogowski A. Blake A. W. Marcus S. E. Gilbert H. J. Knox J. P. (2010). Carbohydrate-binding modules promote the enzymatic deconstruction of intact plant cell walls by targeting and proximity effects. Proc. Natl. Acad. Sci. U. S. A. 107 (34), 15293–15298. 10.1073/pnas.1005732107 20696902 PMC2930570

[B16] Inoue H. Kishishita S. Kumagai A. Kataoka M. Fujii T. Ishikawa K. (2015). Contribution of a family 1 carbohydrate-binding module in thermostable glycoside hydrolase 10 xylanase from *Talaromyces cellulolyticus* toward synergistic enzymatic hydrolysis of lignocellulose. Biotechnol. Biofuels 8, 77. 10.1186/s13068-015-0259-2 26000036 PMC4440266

[B17] Khambhaty Y. Akshaya R. Rama Suganya C. Sreeram K. J. Raghava Rao J. (2018). A logical and sustainable approach towards bamboo pulp bleaching using xylanase from *Aspergillus nidulans* . Int. J. Biol. Macromol. 118, 452–459. 10.1016/j.ijbiomac.2018.06.100 29936081

[B18] Khan M. I. M. Sajjad M. Sadaf S. Zafar R. Niazi U. H. K. Akhtar M. W. (2013). The nature of the carbohydrate binding module determines the catalytic efficiency of xylanase Z of *Clostridium thermocellum* . J. Biotechnol. 168 (4), 403–408. 10.1016/j.jbiotec.2013.09.010 24095983

[B19] Kim J. H. Irwin D. Wilson D. B. (2004). Purification and characterization of *Thermobifida fusca* xylanase 10B. Can. J. Microbiol. 50 (10), 835–843. 10.1139/w04-077 15644898

[B20] Kittur F. S. Mangala S. L. Rus'd A. A. Kitaoka M. Tsujibo H. Hayashi K. (2003). Fusion of family 2b carbohydrate-binding module increases the catalytic activity of a xylanase from *Thermotoga maritima* to soluble xylan. FEBS Lett. 549 (1-3), 147–151. 10.1016/s0014-5793(03)00803-2 12914941

[B21] Kumar S. Arumugam N. Permaul K. Singh S. (2016). Thermostable enzymes and their industrial applications. in Microb Biotechnol: An Interdisciplinary Approach. Editor Shukla P. (FL: CRC Press)115–162. 10.1201/9781315367880-6

[B22] Kumar V. Marín-Navarro J. Shukla P. (2016). Thermostable microbial xylanases for pulp and paper industries: trends, applications and further perspectives. World J. Microbiol. Biotechnol. 32 (2), 34. 10.1007/s11274-015-2005-0 26754672

[B23] Li X. Han C. Li W. Chen G. Wang L. (2020). Insights into the cellulose degradation mechanism of the thermophilic fungus *Chaetomium thermophilum* based on integrated functional omics. Biotechnol. Biofuels 13, 143. 10.1186/s13068-020-01783-z 32817759 PMC7425565

[B24] Li G. Zhou X. Li Z. Liu Y. Liu D. Miao Y. (2021). Significantly improving the thermostability of a hyperthermophilic GH10 family xylanase XynAF1 by semi-rational design. Appl. Microbiol. Biotechnol. 105 (11), 4561–4576. 10.1007/s00253-021-11340-9 34014347

[B25] Lin X. Q. Han S. Y. Zhang N. Hu H. Zheng S. P. Ye Y. R. (2013). Bleach boosting effect of xylanase A from *Bacillus halodurans* C-125 in ECF bleaching of wheat straw pulp. Enzyme Microb. Technol. 52 (2), 91–98. 10.1016/j.enzmictec.2012.10.011 23273277

[B26] Liu S. Shao S. Li L. Cheng Z. Tian L. Gao P. (2015). Substrate-binding specificity of chitinase and chitosanase as revealed by active-site architecture analysis. Carbohydr. Res. 418, 50–56. 10.1016/j.carres.2015.10.002 26545262

[B27] Lombard V. Golaconda Ramulu H. Drula E. Coutinho P. M. Henrissat B. (2013). The carbohydrate-active enzymes database (CAZy) in 2013. Nucleic Acids Res. 42 (D1), D490–D495. 10.1093/nar/gkt1178 24270786 PMC3965031

[B28] Ludwiczek M. L. Heller M. Kantner T. McIntosh L. P. (2007). A secondary xylan-binding site enhances the catalytic activity of a single-domain family 11 glycoside hydrolase. J. Mol. Biol. 373 (2), 337–354. 10.1016/j.jmb.2007.07.057 17822716

[B29] Lykidis A. Mavromatis K. Ivanova N. Anderson I. Land M. DiBartolo G. (2007). Genome sequence and analysis of the soil cellulolytic actinomycete *Thermobifida fusca* YX. J. Bacteriol. 189 (6), 2477–2486. 10.1128/JB.01899-06 17209016 PMC1899369

[B30] Meng D. D. Ying Y. Chen X. H. Lu M. Ning K. Wang L. S. (2015). Distinct roles for carbohydrate-binding modules of glycoside hydrolase 10 (GH10) and GH11 xylanases from *Caldicellulosiruptor* sp. strain F32 in thermostability and catalytic efficiency. Appl. Environ. Microbiol. 81 (6), 2006–2014. 10.1128/AEM.03677-14 25576604 PMC4345376

[B31] Mhiri S. Bouanane-Darenfed A. Jemli S. Neifar S. Ameri R. Mezghani M. (2020). A thermophilic and thermostable xylanase from *Caldicoprobacter algeriensis*: Recombinant expression, characterization and application in paper biobleaching. Int. J. Biol. Macromol. 164, 808–817. 10.1016/j.ijbiomac.2020.07.162 32698070

[B32] Miao Y. Li P. Li G. Liu D. Druzhinina I. S. Kubicek C. P. (2017). Two degradation strategies for overcoming the recalcitrance of natural lignocellulosic xylan by polysaccharides-binding GH10 and GH11 xylanases of filamentous fungi. Environ. Microbiol. 19 (3), 1054–1064. 10.1111/1462-2920.13614 27878934

[B33] Miller G. L. (1959). Use of dinitrosalicylic acid reagent for determination of reducing sugar. Anal. Chem. 31 (3), 426–428. 10.1021/ac60147a030

[B34] Morais S. Barak Y. Hadar Y. Wilson D. B. Shoham Y. Lamed R. (2011). Assembly of xylanases into designer cellulosomes promotes efficient hydrolysis of the xylan component of a natural recalcitrant cellulosic substrate. mBio 2 (6), e00233–11. 10.1128/mBio.00233-11 22086489 PMC3221603

[B35] Motulsky H. (2007). Prism 5 statistics guide. GraphPad Softw. 31, 39–42.

[B36] Pell G. Taylor E. J. Gloster T. M. Turkenburg J. P. Fontes C. M. Ferreira L. M. (2004). The mechanisms by which family 10 glycoside hydrolases bind decorated substrates. J. Biol. Chem. 279 (10), 9597–9605. 10.1074/jbc.M312278200 14668328

[B37] Polizeli M. Rizzatti A. Monti R. Terenzi H. Jorge J. A. Amorim D. (2005). Xylanases from fungi: properties and industrial applications. Appl. Microbiol. Biotechnol. 67 (5), 577–591. 10.1007/s00253-005-1904-7 15944805

[B38] Pollet A. Vandermarliere E. Lammertyn J. Strelkov S. V. Delcour J. A. Courtin C. M. (2009). Crystallographic and activity-based evidence for thumb flexibility and its relevance in glycoside hydrolase family 11 xylanases. Proteins. 77 (2), 395–403. 10.1002/prot.22445 19422059

[B39] Rencoret J. Marques G. Gutiérrez A. Jiménez-Barbero J. Martínez Á. T. del Río J. C. (2013). Structural modifications of residual lignins from sisal and flax pulps during soda-AQ pulping and TCF/ECF bleaching. Ind. Eng. Chem. Res. 52 (13), 4695–4703. 10.1021/ie302810c

[B40] Rio D. C. Ares M. Hannon G. J. Nilsen T. W. (2010). Purification of RNA using TRIzol (TRI reagent). Cold Spring Harb. Protoc. 2010 (6), 5439–5445. 10.1101/pdb.prot5439 20516177

[B41] Ruiz D. M. Turowski V. R. Murakami M. T. (2016). Effects of the linker region on the structure and function of modular GH5 cellulases. Sci. Rep. 6, 28504. 10.1038/srep28504 27334041 PMC4917841

[B42] Saleem M. Tabassum M. R. Yasmin R. Imran M. (2009). Potential of xylanase from thermophilic *Bacillus* sp. XTR-10 in biobleaching of wood kraft pulp. Int. Biodeterior. Biodegrad. 63 (8), 1119–1124. 10.1016/j.ibiod.2009.09.009

[B43] Shi Z. Han C. Zhang X. Tian L. Wang L. (2020). Novel synergistic mechanism for lignocellulose degradation by a thermophilic filamentous fungus and a thermophilic actinobacterium based on functional proteomics. Front. Microbiol. 11, 539438. 10.3389/fmicb.2020.539438 33042052 PMC7518101

[B44] Sonan G. K. Receveur-Brechot V. Duez C. Aghajari N. Czjzek M. Haser R. (2007). The linker region plays a key role in the adaptation to cold of the cellulase from an Antarctic bacterium. Biochem. J. 407 (2), 293–302. 10.1042/bj20070640 17635108 PMC2049020

[B45] Srivastava A. Nagar P. Rathore S. Adlakha N. (2022). The linker region promotes activity and binding efficiency of modular LPMO towards polymeric substrate. Microbiol. Spectr. 10 (1), e0269721. 10.1128/spectrum.02697-21 35080440 PMC8791183

[B46] Talens-Perales D. Sanchez-Torres P. Marin-Navarro J. Polaina J. (2020). *In silico* screening and experimental analysis of family GH11 xylanases for applications under conditions of alkaline pH and high temperature. Biotechnol. Biofuels 13 (1), 198. 10.1186/s13068-020-01842-5 33372612 PMC7720462

[B47] Talens-Perales D. Jimenez-Ortega E. Sanchez-Torres P. Sanz-Aparicio J. Polaina J. (2021). Phylogenetic, functional and structural characterization of a GH10 xylanase active at extreme conditions of temperature and alkalinity. Comput. Struct. Biotechnol. J. 19, 2676–2686. 10.1016/j.csbj.2021.05.004 34093984 PMC8148631

[B48] Tian L. Liu S. Wang S. Wang L. (2016). Ligand-binding specificity and promiscuity of the main lignocellulolytic enzyme families as revealed by active-site architecture analysis. Sci. Rep. 6, 23605. 10.1038/srep23605 27009476 PMC4806347

[B49] Walia A. Guleria S. Mehta P. Chauhan A. Parkash J. (2017). Microbial xylanases and their industrial application in pulp and paper biobleaching: a review. 3 Biotech. 7 (1), 11. 10.1007/s13205-016-0584-6 PMC5385172 28391477

[B50] Wang K. Cao R. Wang M. Lin Q. Zhan R. Xu H. (2019). A novel thermostable GH10 xylanase with activities on a wide variety of cellulosic substrates from a xylanolytic *Bacillus* strain exhibiting significant synergy with commercial celluclast 1.5 L in pretreated corn stover hydrolysis. Biotechnol. Biofuels 12, 48. 10.1186/s13068-019-1389-8 30899328 PMC6408826

[B51] Weiner M. P. Costa G. L. Schoettlin W. Cline J. Mathur E. Bauer J. C. (1994). Site-directed mutagenesis of double-stranded DNA by the polymerase chain reaction. Gene 151 (1), 119–123. 10.1016/0378-1119(94)90641-6 7828859

[B52] Wu X. Tian Z. Jiang X. Zhang Q. Wang L. (2018). Enhancement in catalytic activity of *Aspergillus niger* XynB by selective site-directed mutagenesis of active site amino acids. Appl. Microbiol. Biotechnol. 102 (1), 249–260. 10.1007/s00253-017-8607-8 29103167

[B53] Wu X. Zhang S. Zhang Q. Zhao Y. Chen G. Guo W. (2020). The contribution of specific subsites to catalytic activities in active site architecture of a GH11 xylanase. Appl. Microbiol. Biotechnol. 104 (20), 8735–8745. 10.1007/s00253-020-10865-9 32865611

[B54] Yang J. Han Z. (2018). Understanding the positional binding and substrate interaction of a highly thermostable GH10 xylanase from *Thermotoga maritima* by molecular docking. Biomolecules 8 (3), 64. 10.3390/biom8030064 PMC6163442 30061529

[B55] Yoo C. G. Kim H. Lu F. Azarpira A. Pan X. Oh K. K. (2015). Understanding the physicochemical characteristics and the improved enzymatic saccharification of corn stover pretreated with aqueous and gaseous ammonia. Bioenergy Res. 9 (1), 67–76. 10.1007/s12155-015-9662-6

[B56] Zarafeta D. Galanopoulou A. P. Leni M. E. Kaili S. I. Chegkazi M. S. Chrysina E. D. (2020). XynDZ5: a new thermostable GH10 xylanase. Front. Microbiol. 11, 545. 10.3389/fmicb.2020.00545 32390953 PMC7193231

[B57] Zhao L. Geng J. Guo Y. Liao X. Liu X. Wu R. (2015). Expression of the *Thermobifida fusca* xylanase Xyn11A in *Pichia pastoris* and its characterization. BMC Biotechnol. 15, 18. 10.1186/s12896-015-0135-y 25887328 PMC4369062

[B58] Zheng H. Liu Y. Liu X. Han Y. Wang J. Lu F. (2012). Overexpression of a *Paenibacillus campinasensis* xylanase in *Bacillus megaterium* and its applications to biobleaching of cotton stalk pulp and saccharification of recycled paper sludge. Bioresour. Technol. 125, 182–187. 10.1016/j.biortech.2012.08.101 23026332

