# Supplementary material for: A thermostable and CBM2-linked GH10 xylanase from Thermobifida fusca for paper bleaching
Source: Front Bioeng Biotechnol. 2022 Aug 26;10:939550. doi: 10.3389/fbioe.2022.939550 (PMC9459120; doi:10.3389/fbioe.2022.939550)
Supplement: Supplementary file 1 [file DataSheet1.PDF]

# *Supplementary Material*

**Table S1 The primers sequences of qPCR in this article.**

| Gene name | Gene ID | Primer name | Primer sequence（5′–3′) |
|---|---|---|---|
| *gyra* | AAZ54047.1 | *Tf*GyrA -F1 | ACCTGCTCAGCATGGACGTC |
| | | *Tf*GyrA -R1 | TGTTCGGTGTCTTCGGCGTC |
| | | *Tf*GyrA -F2 | AGCTCGTGGGCGCGCTG |
| | | *Tf*GyrA -R2 | TGCGGGCGATAGCGACC |
| *xyl11a* | AHK22788.1 | *Tf*Xyl11A -F1 | GGCGGAAACTACAGCACCTC |
| | | *Tf*Xyl11A -R1 | TCCGAGGTGCATACCGTGGC |
| | | *Tf*Xyl11A -F2 | GGGGCACCTACCGGCCC |
| | | *Tf*Xyl11A -R2 | GAAGTGGTTCCCCGCGG |
| *xyl10a* | AAZ56956.1 | *Tf*Xyl10A -F1 | CCTGGTACGCGGTCTACGAG |
| | | *Tf*Xyl10A -R1 | AGGTGGCTCCGTTGAGGGTG |
| | | *Tf*Xyl10A -F2 | GCAGGGGGCGATCACCG |
| | | *Tf*Xyl10A -R2 | GCCCGGCTGGAGCGTGG |
| *xyl10b* | AAZ56824.1 | *Tf*Xyl10B -F1 | GGCGACCTGGAACAGAACCT |
| | | *Tf*Xyl10B -R1 | CAGCAGTGATCGTGCTTGGG |
| | | *Tf*Xyl10B -F2 | GCCTGGCCGTGGAAGGC |
| | | *Tf*Xyl10B -R2 | CCCGGCTTGGGGCTGTC |

**Table S2 The primers sequences of *Tf*Xyl10A, *Tf*Xyl10AdC, *Tf*Xyl10A-CD and its alanine mutants.**

| Plasmid name | Primers | Primer sequence（5′–3′) |
|---|---|---|
| *Tf*Xyl10A | *Tf*XylGH10-F | GAGTCGACCCTGCGGGAACT |
| *Tf*Xyl10A | *Tf*XylGH10-R | TCAGGAGCAGGTGGCTCCGT |
| *Tf*Xyl10AdC | *Tf*XylGH10-R2 | TCACGGTTCACCCGGACCGC |
| *Tf*Xyl10A-CD | *Tf*XylGH10-R3 | TCAAGCCTCGTAGACCGCGT |
| E51A | E51A-F | AGATGAAGTGGGCGTCGCTGGAG |
| E51A | E51A-R | CCAGCGACGCCCACTTCATCTCG |
| Q89A | Q89A-F | CTGGCACAGCGCGCTGCCGTCCT |
| Q89A | Q89A-R | CTGGCACAGCGCGCTGCCGTCCT |
| K49A | K49A-F | ACGAAAACGAGATGGCGTGGGAGTC |
| K49A | K49A-R | GACTCCCACGCCATCTCGTTTTCGT |
| N46A | N46A-F | CCCACGAAGCCGAGATGAAGTGGGA |
| N46A | N46A-R | TCCCACTTCATCTCGGCTTCGTGGG |
| W276A | W276A-F | GCATCGGTATCACCGTCGCGGGTAT |
| W276A | W276A-R | ATACCCGCGACGGTGATACCGATGC |
| E45A | E45A-F | TCACCCACGCAAACGAGATGAAG |
| E45A | E45A-R | CTTCATCTCGTTTGCGTGGGTGATG |
| Q206A | Q206A-F | TCCACGGTATCGGTATCGCGTCCC |
| Q206A | Q206A-R | GGGACGCGATACCGATACCGTGGAT |
| H208A | H208A-F | GTATCCAGTCCGCCCTGATCGTCG |
| H208A | H208A-R | CGACGATCAGGGCGGACTGGATAC |
| H82A | H82A-F | CGGCGCCACCCTGGTCTGGCA |
| H82A | H82A-R | TGCCAGACCAGGGTGGCGCCG |
| N130A | N130A-F | GGTCGCCGAAGCGTTCAACGAGG |

| | | |
|---|---|---|
| N130A | N130A-R | CCTCGTTGAACGCTTCGGCGACC |
| N172A | N172A-F | CATCGCCGACTACAACATCGAAGG |
| N172A | N172A-R | CCTTCGATGTTGTAGTCGGCGATG |
| W86A | W86A-F | GGTCGCGCACAGCCAGCTGCC |
| W86A | W86A-R | CAGCTGGCTGTGCGCGACCAG |
| W284A | W284A-F | GCTCCGCGGTGCCCTACACCTTC |
| W284A | W284A-R | AAGGTGTAGGGCACCGCGGAGCG |
| E131A | E131A-F | TCAACGCGGCGTTCAACGAGGAC |
| E131A | E131A-R | GTCCTCGTTGAACGCCGCGTTGA |
| E236A | E236A-F | ATCACCGCGCTGGACATCCGCAT |
| E236A | E236A-R | ATGCGGATGTCCAGCGCGGTGAT |
| Y174A | Y174A-F | ACGACGCCAACATCGAAGGCATCA |
| Y174A | Y174A-R | TGATGCCTTCGATGTTGGCGTCGT |
| E135A | E135A-F | GTTCAACGCGGACGGTACGCTC |
| E135A | E135A-R | TACCGTCCGCGTTGAACGCTTCG |
| N175A | N175A-F | ACGACTACGCCATCGAAGGCATCA |
| N175A | N175A-R | TGATGCCTTCGATGGCGTAGTCGTT |

**Table S3 Specific activities of *Tf*Xyl10A, *Tf*Xyl10AdC and *Tf*Xyl10A-CD.**

| Enzyme | Specific activity (U/mg) | | |
|---|---|---|---|
| | On BX | On PCS-1 | On PCS-2 |
| *Tf*Xyl10A | 368.86±28.85 | 106.05±0.88 | 60.49±1.84 |
| *Tf*Xyl10AdC | 450.50±23.83 | 102.71±1.66 | 33.68±1.96 |
| *Tf*Xyl10A-CD | 145.27±8.97 | 54.14±1.72 | 40.93±3.84 |

**Table S4 Residual activities of supernatant of *Tf*Xyl10A, *Tf*Xyl10AdC, and *Tf*Xyl10A-CD after binding with insoluble xylan and microcrystalline cellulose (MCC) at 4 °C for 1 h.**

| Enzyme | Residual activity (U/mg) | |
|---|---|---|
| | after binding with insoluble xylan | after binding with microcrystalline cellulose (MCC) |
| *Tf*Xyl10A | 288.89±29.34 | 25.54±8.23 |
| *Tf*Xyl10AdC | 455.98±25.68 | 267.35±37.7 |
| *Tf*Xyl10A-CD | 105.18±2.16 | 100.93±6.14 |

**Table S5 Specific activities of WT (*Tf*Xyl10A-CD) and its variants on BX.**

| Enzyme | Specific activity (U/mg) | Relative activity (%) |
|---|---|---|
| WT | 145.27±8.97 | 100±4.48 |
| E51A | 290.61±3.33 | 190.43±2.19 |
| Q89A | 46.18±2.12 | 30.26±1.39 |
| K49A | 62.78±3.95 | 41.14±2.59 |
| N46A | 20.89±3.29 | 13.69±2.16 |
| W276A | 16.00±0.23 | 10.48±0.15 |
| E45A | 34.74±7.22 | 22.77±4.73 |
| Q206A | 74.61±4.26 | 48.89±2.79 |
| H208A | 9.62±0.84 | 6.31±0.55 |
| H82A | 11.90±1.49 | 7.80±0.98 |
| N130A | 68.44±3.72 | 44.85±2.44 |
| N172A | 49.45±9.50 | 32.40±6.22 |
| W86A | 25.61±2.96 | 16.78±1.94 |
| W284A | 21.10±1.39 | 13.83±0.91 |
| E131A | 15.66±1.36 | 10.26±0.89 |
| E236A | 10.12±0.41 | 6.63±0.27 |
| Y174A | 14.06±3.12 | 9.22±2.04 |
| E135A | 42.36±6.90 | 27.76±4.52 |
| N175A | 126.46±4.43 | 82.87±2.90 |

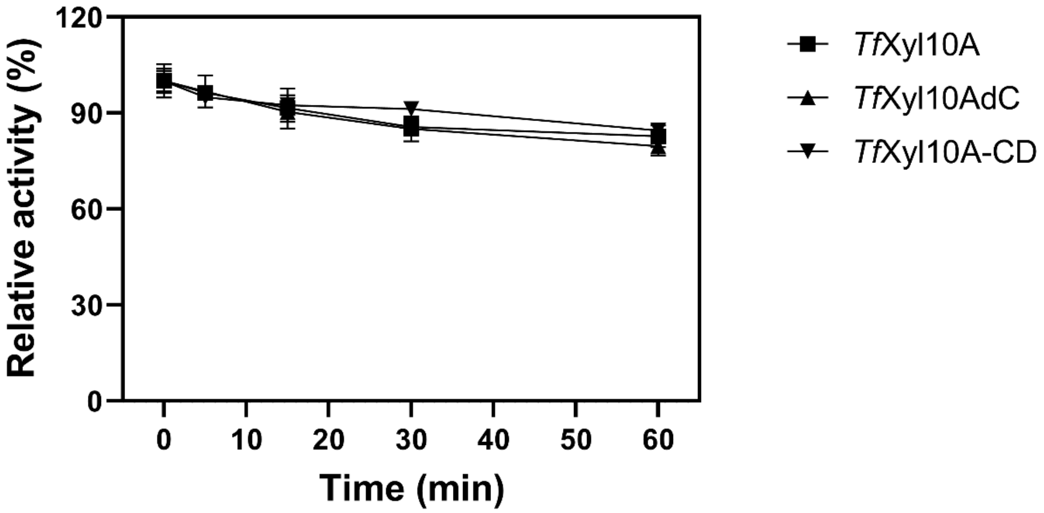


**Figure S1 pH stabilities of three xylanases at pH 9.0 under different time points (0, 5, 15, 30, and 60 min). The activity of the untreated sample was set to 100%. The bars indicate the standard errors.**

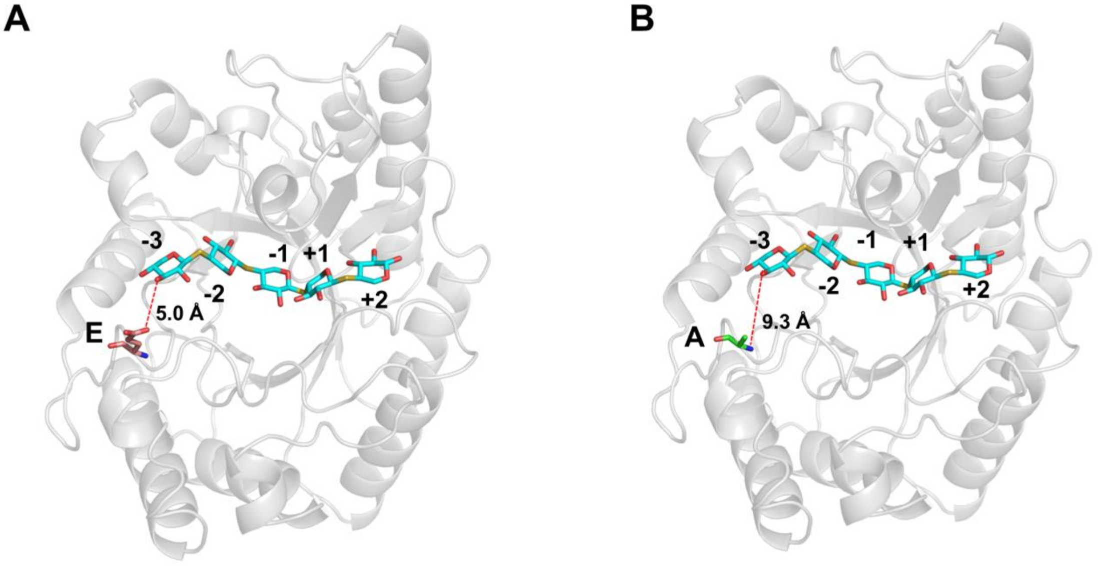


**Figure S2 The distance between the amino acid located at 51 and substrate of wild-type enzyme (A) and E51A mutant (B)**.
